# Translating tools and indicators in territorial RRI

**DOI:** 10.3389/frma.2022.1038970

**Published:** 2023-01-09

**Authors:** Thomas Völker, Marzia Mazzonetto, Rasmus Slaattelid, Roger Strand

**Affiliations:** ^1^Centre for the Study of the Sciences and Humanities, University of Bergen, Bergen, Norway; ^2^BE Participation, Brussels, Belgium

**Keywords:** RRI, translation, care, indicator politics, responsibility, SwafS, Responsible Research and Innovation, regional innovation

## Abstract

**Introduction:**

By a series of calls within the Horizon 2020 framework programme, the EU funded projects intended to deploy Responsible Research and Innovation (RRI) at a territorial level, in regional research and innovation ecosystems. This paper presents efforts to document and evaluate the achievements in TRANSFORM, one of these projects.

**Methods:**

Evaluative inquiry and theoretical reasoning.

**Results:**

Noting the need for a general principle to be interpreted, adapted and translated in order to be rendered meaningful at a local level, we studied precisely these multiple territorial translations of RRI, the organizational and institutional orderings with which they co-emerge and the challenges that come with these translations. An important shared feature is that RRI work does not start from zero, but rather builds on pre-existing relationships and repertoires of collaboration. The RRI project is hence a way to continue ongoing work and follow pre-set purposes, aims and objectives, as a form of “maintenance work”. In this very human sense, RRI is deployed with a logic of care in the regional context, while the Horizon 2020 calls and proposals above all are formulated in a logic of choice, to be assessed by indicators.

**Discussion:**

We warn against undue standardization of RRI by toolification and use of quantitative indicators, and recommend that RRI performance is monitored by methods of evaluative inquiry.

## Introduction

“*No object considered purely in and for itself, in terms of its intrinsic attributes alone, can be a tool. To describe a thing as a tool is to place it in relation to other things within a field of activity in which it can exert a certain effect. Indeed we tend to name our tools by the activities in which they are characteristically or normatively engaged, or by the effects they have in them. Thus to call an object a saw is to position it within the context of a story such as the one I have just told, of cutting a plank. To name the tool is to invoke the story. It follows that for an object to count as a tool it must be endowed with a story, which the practitioner should know and understand in order to recognise it as such and use it appropriately. Considered as tools, things are their stories.” (Ingold*, *2011**, 56)*.

The beauty of this quote from social anthropologist Tim Ingold lies in the fact that he takes the most toolish of tools—a mundane saw—and casually proceeds to deny it its essentialist qualities as a saw. Instead, he argues that what lends the saw its “sawiness” are not inherent qualities or attributes but narratives and relations. Stories about what the saw does or what is done with it and about the relations it creates. The argument we want to develop in this paper then is the following: what is true for a saw, also has value for thinking about research and innovation practices and ecosystems.

During the last decade Responsible Research and Innovation (RRI) has gained some traction both in academic as well as in policy discussions. RRI grew out of policy debates on innovation governance and resonates with longstanding concerns about changing ways of producing and circulating knowledge (von Schomberg, 2012). The concept relates to earlier debates in academia and policy diagnoses of the changing relations between science, society, politics and innovation (Owen et al., 2012; Bauer et al., 2021). RRI also aims to find more socially robust ways of assessing and governing emerging technologies.

RRI gained further traction in the Horizon 2020 “Science with and for Society” (SwafS) programmes. Here the idea of a cultural shift in our collective ways of engaging with science, technology and innovation—and the objective of creating stewardship for our shared futures, developed into an endeavor of creating tools for implementing RRI on a local level.

We are interested in precisely these multiple territorial translations of RRI, the organizational and institutional orderings with which they co-emerge and in the challenges that come with these translations. Furthermore, we are interested in translation processes that are connected to the implementation of RRI as a set of tools (e.g., for engagement of citizens in regional development and awareness raising projects). In this paper we present a concrete case to begin to explore the following questions:

- How is RRI translated from the transnational context of EU policymaking to national and regional contexts and across different sectors?
- What are the consequences of conceiving RRI as a set of tools to be deployed within regional research and innovation stories/narratives?
- How should the performance of RRI, as tools in territorial contexts, be evaluated?

These questions will be explored in the context of an empirical investigation of RRI pilot projects, from a project funded through the Horizon 2020 SwafS programme. We further bring together work from the field of Science and Technology Studies (STS) with approaches from evaluations studies and practice in order to answer these questions.

### From engagement to RRI and back again

When discussing present and future translations of RRI, it is useful to recall how RRI itself can be seen as a translation of earlier concepts and developments. Already back in the 1990s different modes of governance and new relations between actors that are (and should be involved) were discussed in both academic and policymaking circles. Mode 2 and Post-Normal Science were two of the conceptual innovations in these discussions, both arguing that the role of science in society was changing and that non-academic actors have an important part to play in knowledge production (Funtowicz and Ravetz, 1993; Gibbons, 1994; Nowotny et al., 2001). Closely related to these debates, the concept of the triple helix (Etzkowitz and Leydesdorff, 1998) attracted some interest in debates about changing science-society relations—university-industry-government relations to be precise. More recently, notions of a quadruple or even quintuple helix gained some momentum, adding publics and the environment to the helical structure (Mehari et al., 2022).

In addition, RRI grew out of a body of work concerned with technology assessment (Guston and Sarewitz, 2002; Rip and Kulve, 2008) and anticipatory governance (Barben et al., 2007; Guston, 2013; Nordmann, 2014) with anticipation still being one of the key pillars of RRI. Research on the Ethical, Legal and Social Aspects or Implications (depending on which side of the Atlantic you happen to live and work) is considered to be the direct predecessor of RRI (Fitjar et al., 2019; Strand, 2019). While the establishment of these notions and the related practices are considered to be important milestones in the governance of technoscientific innovation, there was often a feeling of unease when these reflections were relegated to separate work packages in projects or toward the end of innovation processes. RRI was supposed to move “upstream” (Krabbenborg and Mulder, 2015).

On a policy level, ideas of responsible modes of conducting and governing research and innovation can be traced back at least to 2001, when the White Paper on European governance (COM(2001) 428) laid out a set of principles for good governance: openness, participation, accountability, effectiveness and coherence. This document argued that “participation crucially depends on central governments following an inclusive approach when developing and implementing EU policies” (ibid., 8). At that time, European legislation on the environment, health and safety also stressed the importance of broadening the set of actors that were considered relevant and intended to give more agency to European citizens. The Environmental Impact Assessment Directive (85/337/EEC; amended subsequently by Directive 2003/35/EC), the Directive (96/82/EC) on the control of major-accident hazards involving dangerous substances, and the Water Framework Directive (2000/60/EC) are examples of such ambitions. These documents are important for framing the debate on governance and participation also because of the ways in which citizens are conceptualized. Such framings in legal documents determines who is entitled to participate and also what can be legitimately expected from these engagements. Some examples of this are Article 14 of the Directive 2000/60/EC, which mentions a “concerned public” and “users” while Directive 2012/18/EC in Articles 14 and 15 talks about “the public affected or likely to be affected by, or having an interest in, the decision making on any of the matters covered by Article 15(1).”

While these debates circled around ideas of participation in relation to accountability and transparency, the notion of responsibility itself gained traction on a policy level around 2011 through a series of high-level expert workshops. In 2014 the Directorate General for Research (DG Research) of the European Commission established RRI as a cross-cutting issue in its research and innovation funding programme Horizon 2020. The framing of responsibility in this discourse is nicely exemplified in the Rome Declaration:

“*RRI requires that all stakeholders including civil society are responsive to each other and take shared responsibility for the process and outcomes of research and innovation. This means working together in: science education; the definition of research agendas; the conduct of research; the access to research results; and the application of new knowledge in society – in full respect to gender equality, the gender dimension in research and ethics considerations*.^1^”

Responsiveness and collaboration across institutional boundaries are core concerns for RRI as expressed in this quote. Crucially, this is a responsibility that needs to be shared throughout the whole process. It is furthermore important to share responsibility in the aftermath of the process, when the outcome of said processes have become clear. This points to the idea of upstream engagement in research and innovation, or as Stilgoe et al. (2013, p. 1570) put it “taking care of the future through collective stewardship of science and innovation in the present.” This notion of responsibility thus relates to work on the notion of care (Puig de la Bellacasa, 2011, 2017; Felt et al., 2013; Halpern et al., 2016). The crucial point in using this notion is that logics of choice that are still dominant in research and innovation are contrasted with a logic of care that focuses on engaging in long-term collaborations addressing matters of concern (Latour, 2004).

This crucially also relates to the governance of emerging scientific fields and technological innovations. RRI is then understood as a new mode of governance necessary for addressing “ethically problematic” (Owen et al., 2012, p. 751) areas such as genetically modified organisms or synthetic biology. Therefore, Owen and his colleagues claim that science no longer can be content with being “in” society. Science needs to produce knowledge “for” society “with” society (ibid.). Stilgoe et al. (2013) introduce a set of principles regarding responsible procedures where anticipation, reflexivity, inclusion and responsiveness are key. Ideas present in previous discussions outlined above clearly resonate in such principles: “anticipation” for example takes a critical stance toward top-down risk assessment and calls for focusing on the social, ethical, and political stakes related to technoscientific developments, while calls for inclusion and responsiveness stress the necessity of participatory modes of governance and collaborative modes of knowledge production. This is not surprising since this work is explicitly situated within a long line of scholarly debate and policy discussion that Ulrike Felt has called a “stratigraphy of science-society policies” (Felt et al., 2013, p. 13), in which different layers of thinking about science-society relations, relevant actors and modes of engagement get “sedimented” instead of completely replacing each other.

Coming back to the quote from Tim Ingold we started out with, what becomes visible already in this brief and incomplete summary of debates on responsibility in science, research and innovation, is that traces of stories emerge when items in the RRI toolbox are placed in relation to other things within a field of activity (Mode 2, PNS, Triple Helix, TA, etc.) in which it exerts a certain effect. These stories may teach us something about what kind of work RRI tools do. In several current RRI projects funded through the Horizon 2020 SwafS programme, we see a (re-)focusing on public participation and citizen engagement tools, ranging from citizen science activities, to attempts to establish citizen assemblies. The debate on responsible research and innovation thus in a sense comes full circle. What started out as debates about new modes (tools) of governance and technology assessment through the inclusion of extended peer communities and then developed into a broad range of “keys” (tools) for responsible (innovation) policymaking, thus seems to return to its core principles and ambitions, namely public engagement and, through that, the ethical challenge raised by the changing social contract of science (Bauer et al., 2021).

### RRI as translation(s) in ecologies of participation

We introduced this paper with a quotation from Tim Ingold on the understanding of tools as stories, and we have suggested that the analogy between concepts and artisanal tools that is already established in RRI^2^ may be methodologically productive in understanding the effects of RRI. This requires understanding the limits of the analogy. With its definition(s), “keys” and “conditions,” RRI consists of a conceptual framework on the basis of which conceptual tools in the form of workshop design principles, decision making games, public engagement protocols, and the like can be constructed to help achieve more responsible research and innovation. Inspired by Ingold's definition of the tool as being its story, we follow the use of a set of RRI tools in three distinct EU regions. The act of implementing these general tools in concrete local contexts can be seen as examples of translation.

In linguistics the term refers to the act of converting a text or a word from one language to another, or the result of such a shift. The term however is also used in other senses not restricted to language. Inspired by Michel Serres and Michel Callon, Bruno Latour uses translation in a non-linguistic sense to mean “displacement, drift, invention, mediation, the creation of a link that did not exist before and that to some degree modifies two elements or agents.” (Latour, 1994) The way, for instance, in which the relationship between a human and a tool creates a link that may modify the purposes and goals of both the tool and the human. More recently, the idea of translation has gained some traction in the assessment of engagement activities. Studies of this kind are especially interested in the “travel” of standardized methods or tools for engagement (Soneryd, 2015; Soneryd and Amelung, 2016; Laurent, 2017; Konopásek et al., 2018).

Linda Soneryd's uses the term “translation” in her analysis of engagement activities, and the paper at hand will build on her understanding of the concept. Her work brings together a material-semiotic notion of translation with organizational sociology and focuses on how “technologies of participation” (Soneryd, 2015) get transformed in their practical application in different local settings. She starts from the insight that engagement tools, techniques and methods are not stable entities but instead are continuously re-shaped:

“*When public participation instruments are situated in specific local contexts, however, their ideas, values, formal rules, and tools become remixed, giving rise to new meanings.” (2016: 171)*.

The way she uses translation the focus is on both (1) the shifts—“re-mixes”—in meaning of concepts like participation, citizen, expert and so on, and (2) the making and re-making of links between different actors thus directing attention to the political and organizational settings in which they are applied. The main advantage of this understanding of the term is that it doesn't assume an essence of RRI. Instead, RRI becomes a relational concept in the sense that it undergoes shifts in meaning in relation to other things in the field of activity where it is placed, leading to a displacement and a modification of the concept/tool as well as the new surroundings. This is also expressed in John Law's definition of the term as the combination of “traduction/trahison,” of similarity and difference (Law, 2003) or in Andrew Barry's way of thinking about translation as a process of replication through imitation and differentiation (Barry, 2013).

Translation focuses on both similarity and difference simultaneously and thus directs our empirical attention to the regional specificities when applying tools that are to some extent standardized on different scales. Such a relational understanding of RRI and its tools allows us to focus on how it is practiced in different cases of RRI application. Translation provides a fruitful way to understand how exactly ideas—or policy concepts like RRI—translate and materialize in ever new forms.

What is furthermore interesting and resonating with our work in the TRANSFORM project is Soneryd's call for research into the performativity of engagement discourses. As she puts it,

“*we need to treat this growing interest in public engagement instruments as a research object in its own right and potentially as a new organized space that changes the conditions for governance.” (2016: 157)*.

This points to questions about the work that RRI discursive practices and translations are doing in different settings, who is doing this and with what consequences. The organizational settings of individual engagement activities are addressed through a focus on organizational carriers (organizations and networks) and normative and symbolic systems (e.g., shared beliefs and unquestioned dogma) as visible in ideological frames or institutional “myths.”

This framing is closely tied to a co-productionist approach to the evaluation of participation and engagement processes captured in the notion of “ecologies of participation” (Chilvers and Kearnes, 2015; Chilvers et al., 2018). Jason Chilvers and Matthew Kearnes have adopted this conceptual framework, which states that “the ways in which we know and represent the world (both nature and society) are inseparable from the ways in which we choose to live in it” (Jasanoff, 2004, 2), to the analysis of engagement and participatory initiatives. They conceptualize engagement activities as

“*contingent and heterogeneous collectives of human and non-human actors, devices, settings, theories, social science methods, public participants, procedures and other artefacts.” (Chilvers and Kearnes*, *2015**, 15)*.

The different elements in this quote are understood as mutually constitutive. This means that certain ideas or enactments of publics are connected to the issues that are debated, to the cultural and political settings within which these debates take place and—crucially—to the particular engagement “tools” through which they are addressed. Engagement from such a relational, co-productionist perspective focuses on how engagement and participation is practiced as a part of techno-political orderings. What this means is that the analytical focus moves away from how accurate certain methods are implemented and instead asks for the material organizational-institutional settings, scientific knowledge claims, engagement objects, issues at stake, subject positions and (collective) identities that shape the engagement practices. Such a relational understanding also stresses the connections between different actors and organizations (with their respective conventions and ideas about engagement) involved in these activities—or what they call “engagement collectives.” To capture such multiple connections between different collectives and practices Chilvers and Kearnes use the metaphor of “ecology”:

“*An ecological conception of participation suggests that is not possible to properly understand any one collective of participation without understanding its relational interdependence with other collective participatory practices, technologies of participation, spaces of negotiation and the cultural-political settings in which they become established.” (Chilvers and Kearnes*, *2015**, 52)*.

This focus on what Chilvers and Kearnes call “ecologies” is deliberately comparative in nature as it stresses the importance of staying attentive to the relation between different engagement collectives and to how they become part of a broader political or democratic culture.

Framing the analysis in these terms then means understanding participatory practices as part of particular organizational settings and issue spaces, as well as understanding these practices as embedded in certain legal frameworks, infrastructures, collective imaginaries, established social practices, and collective forms of public reason (Jasanoff, 2003; Jasanoff and Kim, 2015; Chilvers et al., 2018).

Hence, we see a double movement of translation, as RRI is translated in specific ways in different cases, be it through different methods, approaches, or tools. This in turn suggests that these methods are translated as RRI in specific ways. Importantly, these shifts and changes are not random. They are entwined with the political and organizational contexts in the three different cases we present in the empirical section of this paper. Looking at RRI practices through this conceptual lens sensitizes us to how various methods or tools are translated in or into localized research and innovation settings (instead of focusing on the creation of scalable and transferable tools for RRI projects). How does RRI travel and how are the core ideas of RRI re-shaped in the process?

### Evaluating responsibility and the entry of indicator politics

Before we turn to the empirical section of this paper and explore territorial translations of RRI in three European regions, it will be useful to clarify one aspect of what could be called the translational machinery, or rather one dimension of the particular institutional context in which the translations occur. The dimension we refer to, is the fact that they occur within a project funded by the SwafS programme of Horizon 2020, that is, as activities funded by and defined in negotiation with the European Commission as a contractual partner.

As might be expected, RRI scholarship is highly reflexive. There is an abundance of journal papers and book chapters where RRI scholars reflect on the theory and practice of RRI, including their own experiences and practices. There is a great diversity of understandings of responsibility (Christensen et al., 2020). Several authors juxtapose academic or scholarly understandings of RRI with understandings and practices in the policy domain, in particular within the EU/EC (Rip, 2016; Burget et al., 2017), sometimes denoted aRRI (for academic RRI) and pRRI (for policy RRI), respectively (Klaassen et al., 2018). In these juxtapositions, these authors have tended to describe the tensions between the exploratory, experimental and learning-oriented aRRI and the more managerial and outcome-oriented pRRI, typically with a normative preference for the former.

We propose that the relationship between aRRI and pRRI can also be seen as one of translational processes. Going beyond the scope of this paper, we would suggest that these processes have been bidirectional and dialectical in their nature. Focusing on the context of SwafS projects, however, it is easily seen that the institutionalization of RRI initiatives into European Commission work programmes and grant agreements has implications on their form and content. RRI grew out of understandings of good governance that emphasized openness, learning and participation. On the other hand, the public administration of EU's framework programmes, as developed in DG RTD and later its dedicated European Research Executive Agency, has to conform to EU standards of accountability and effectiveness, and, we would argue, in practice has come to emphasize those two dimensions of good governance and could be seen as an instance of a strong audit culture.

It is in this context that the question of performance arises: How should the performance of RRI be evaluated? Within the policy context, the legitimacy of this question is self-evident. One cannot defend expenditures of public money without somehow measuring or monitoring the effectiveness of the funded activities. Also scholars within a typical aRRI discourse have argued for the reasonableness of defining success criteria and performance indicators for RRI (Wickson and Carew, 2014; Yaghmaei and van den Poel, 2020). However, the tension in how to understand the content of RRI—summarized in the aRRI/pRRI-distinction—was reproduced in the debates on how to evaluate RRI, even within the attempts taken by DG RTD to clarify the issue. Hence, in the mid-2010s, DG RTD created two parallel initiatives to answer the question of how to monitor and evaluate RRI, namely the Expert Group on Policy Indicators for RRI^3^, and a tendered project called MoRRI: Monitoring the evolution and benefits of RRI. Both initiatives got a mandate to develop indicators within the prevailing audit culture of the EC, as specified by the so-called SMART principles that called for indicators to be specific, measurable, attainable, relevant and timely. The expert group largely evaded the demand for SMART, argued for a network approach to governance, and mostly proposed qualitative RRI indicators suitable for processes of self-governance (European Commission Directorate-General for Research Innovation, 2015; see also Strand and Spaapen, 2020). The MoRRI project took the other route and devised mostly quantitative indicators, arguing:

*The inability to evaluate, compare and benchmark constitutes a barrier to international and organisational learning, whereas identification of useful indicators and metrics for RRI might contribute to bringing RRI from a peripheral position closer to the centre of activity*. (Fochler and de Rijcke, 2017, p. 7).

The EC never implemented any of the indicators of the expert group. The MoRRI indicators, however, were gradually introduced into the SwafS work programmes to the extent that expected impact of SwafS projects were defined in terms of them. This was also the case for the TRANSFORM project that is the empirical substrate of this paper. In the so-called SwafS-14 calls for proposals for territorial RRI actions, the description of expected impact ended as follows: “Consortia are expected to contribute to one or more of the MoRRI indicators (for instance GE1, SLSE1, SLSE4, PE1, PE2, PE5, PE7, PE8, E1, OA6, GOV2), and to the Sustainable Development Goals (for instance goals 4, 5, 9, 11, 12, 13, 16 or 17).^4^” As an example, the GE1 MoRRI indicator is numerical and defined as “share of research-performing organizations with gender equality plans.” GOV2 can be either qualitative or quantitative and is defined as “RRI-related governance mechanisms within research-funding and performing organizations.”

For researchers working within SwafS actions, the presence of MoRRI indicators has been somewhat of a headache, to the extent that SwafS-funded projects together with MoRRI's successor, SUPER MoRRI, have met regularly over years to discuss the methodological challenges involved with them.^5^ The headache is not the least caused by the fact that critiques of audit culture and what has been called “trust in numbers” (Porter, 1995) are part of the intellectual origins of RRI as outlined above. The popularity of quantification for governance is usually attributed to the ability of numbers to “travel” and thus to work as a technology for knowing and governing at a distance (Latour, 1987; Scott, 1998). RRI, on the other hand, can be seen as part of a counter-movement against technocracy. RRI scholars know that all indicators are necessarily political and that their introduction in itself causes translations of practice. As Merry puts it: “The technical is always political because there is always interpretation and judgement in systems of classification, in the choice of things to measure, in the weighting of constitutive elements, and in decisions about which denominator to use for a ratio. The political hides behind the technical.” (Merry, 2016: 21). The development of indicators is not merely about the neutral measurement of an already pre-existing object “out-there” but rather involves the production of an object as legible and governable (Völker et al., 2020). By this we mean that the way in which an entity like responsibility is quantified and measured is far from being self-evident.

In sum, the seemingly innocent question of how to evaluate responsibility is intrinsically entangled into the question of how to translate responsibility. For the authors of this paper, the question of how to evaluate, and the very practical question of how to deal with our contractual obligations to deal with MoRRI indicators, was the very starting point for this paper. Thinking about indicator politics in relation to responsible research and innovation entails taking a reflexive stance toward the performativity of the indicators that are chosen to measure and assess responsibility in different projects. This means asking questions about the kinds of work that are made visible through particular forms of quantification and the kinds of work that get marginalized. Indeed, in what follows, we shall describe translations of RRI that in our view constitute real achievements and add value. However, these achievements would largely be made invisible by the stringent use of MoRRI indicators.

## Materials and methods

In this paper we present a comparative analysis of three different RRI pilot projects within the Horizon 2020-funded project TRANSFORM. The project describes itself like this:

“*The EU-funded TRANSFORM project is putting RRI principles into practice. It brings together three European regions – Lombardy (Italy), Brussels-Capital (Belgium) and Catalonia (Spain) – to design, test and disseminate three sound co-creation methodological frameworks (participatory research agenda setting, design for social innovation and citizen science) within their Smart Specialisation Strategies (S3). The three implementing regions will engage in mutual learning within and beyond Europe, pairing with a co-design initiative in Boston (USA). The objective is to establish more open, transparent and democratic R&I ecosystems for more responsible territorial development*.^6^”

In the pilots that are developed in the three different regional clusters RRI takes the shape of citizen science projects, design thinking, and participatory agenda setting and (plans for a) citizen assembly. Thus, we see different translations of RRI steered by diverse actors and embedded within different R&I ecosystems.

The analysis builds on material gathered in the project TRANSFORM. The core material consists of interview data from 12 semi-structured interviews (Lamont and Swidler, 2014) with 15 project partners working in the different territorial RRI pilots of the TRANSFORM project. The interview guide consisted of the following core themes:

(1) The activities in the RRI pilot projects and the different purposes and rationales that are guiding this work.
(2) The specific (systemic) hurdles and resistances that our colleagues are facing in their work. In this section we also addressed the institutional-political context of the different TRANSFORM pilot projects.
(3) Experiences with previous attempts of doing RRI and RRI-like work in the region (and how the current activities relate to those).
(4) Reflections on what might come after the project's activities: their continuation, legacy, and impact.

The interviews lasted between 60 and 120 min and were transcribed and coded according to Srivastava and Thomson (2009). In addition to that, project documentation and relevant policy documents have been analyzed using the same type of coding framework.

This paper carves out a range of different translations of RRI in territorial pilot projects in the three TRANSFORM clusters in Lombardy, Catalonia and the Brussels-capital region while also directing attention to the organizational and institutional ecosystem that both enables the pilot projects' work and further shapes how it plays out in practice.

In our work we take inspiration from recent evaluation approaches that focus on “indicating” (Marres and de Rijcke, 2020) and “evaluative inquiry” (Fochler and de Rijcke, 2017):

“*‘Evaluative inquiries' are not solely structured along the lines of externalizing explanations and metrics. They are also capable of representing the heterogeneous associations and practices that constitute our work. (…) Evaluative inquiries perform a shift from a predominantly bureaucratic to more substantive modes of assessment. In this, a standardization of indicators and methods is less relevant than “staying with the trouble” (Haraway 2016); staying closer to the epistemic missions, frictions and resonances of the work under scrutiny.”* (Fochler and de Rijcke, 2017, p. 34).

This approach aims to represent complexity and further attempts to describe in detail different missions and frictions and how they are co-emergent with different social, epistemic, normative and organizational orderings. When thinking about indicators and tools for RRI, the objective then becomes to understand RRI tools and methods as situated in and mutually constitutive with particular institutional-organizational settings.

### Empirical analysis

“*I say RRI because it's basically a big, for me/ well I know this is recorded but for me RRI is big, how can I say. Like, it's like a big bag where I put a lot of stuff (laughter) in it. But it's all about me being more open, more transparent, involving more the society and I know that there is also the question about ethics and yeah, openness of data and all these things.”*

In the empirical section of this paper, we will take a look at different translations of RRI. We will do so by exploring a range of regional RRI projects within the broader framework of the TRANSFORM project.

The quote above nicely illustrates how actors from the pilots tend to understand RRI stressing the ambiguous nature of the term by comparing it to a bag that can accommodate different things designated by the same collective term. Openness, transparency, the inclusion and engagement of a broad range of actors, ethics, open data, etc. The bag can also be read as a translation of the toolkit/toolbox metaphor which is an established image in RRI discourse.

Throughout this section we will revisit this ambiguity and the pluralism it enables. We will not go into details about the various RRI activities, but rather address some of the broader themes that emerged in the conversations and in the additional materials we looked at.

We will start by (1) showing how the very idea of an RRI pilot and the (imagined) purposes of such a pilot gets translated in different ways in the different RRI pilot projects we looked at. Following that we will (2) zoom in on the actors' own stories about the legacy of these pilot activities and the kinds of work that enable and sustain such legacies. Finally, we will (3) take a look at how the broader notion of acceptance is translated in the activities in the clusters and the tensions this might create.

### What is a pilot—Imagined purposes of RRI

The activities in the different regional project clusters are organized as pilots. The Lombardy cluster developed and conducted a participatory research agenda setting process focused on regional innovation strategies and a citizens' jury on smart mobility. These activities are collaboratively developed by Fondazione Giannino Bassetti (FGB) and their partners from the regional administration, Regione Lombardia and Finlombarda. Similar to this cluster, also the work of our Catalan colleagues builds on a strong collaboration between the research and administrative partners. One of the two Catalan pilots aims at improving regional waste collection systems. This is done through a citizen science approach working with secondary school pupils in the suburban town Mollet del Vallès. In addition, departments of the municipality are involved. An interactive digital waste game was designed and subsequently used with the assistance of secondary school students. The second pilot of the Catalan cluster addresses the issue of endometriosis and aims at improving services for the diagnosis, care and support in relation to the disease. This pilot also translates RRI as citizen science and managed to set-up a collaboration between patients, medical staff at Hospital Sant Pau in Barcelona as well as the Catalan Agency for Health Quality and Assessment. The Brussels-Capital Region (BCR) cluster is conducting two distinct pilot activities. One of these pilots is dealing with the issue of unsold food while the other one involved two students of the Catholic University of Louvain with their projects in the broader area of the circular economy, specifically on the development of water sensors (in a project called “AcquaSens”) and in the broader area of circular foods (called “Algorella”). In these pilots RRI is translated as an urban development project relying on co-creation methods in the unsold food case, and as quadruple helix engagements following a design thinking approach in the cases of AcquaSens and Algorella.

What is notable with regard to the question of translation is that the term “pilot” means different things in the different clusters and even for different partners within the clusters. There are certain objectives, purposes and potentials associated with such pilots as well as different risks. A pilot activity can for example entail designing an activity and “piloting” it in the sense of tinkering and experimenting with it. The goal is to fine-tune the approach and methodology. In contrast, a pilot can also be something—and this is more outcome focused—that can be used as a piece of evidence or exhibit in a process of proofing the “added value” of RRI.

“*She's very sceptical about these types of processes being useful to all type of research and innovation. […] I told her let's, just give us the opportunity and so let's do one small experiment and see and then it's up to you to, to judge. I mean I'm not of course trying to preach anything here. So she said no let's do it the other way around. You show me the added value and if I believe in it, if I get convinced by it then I can open doors for you.”*

The objective of one of the pilot projects in the BCR cluster was to present the added value of mainstreaming RRI approaches at a regional funding agency. This, then, is a story about an RRI-pilot as a means for building relations to and within the regional R&I system, and about how innovation projects are set up and evaluated (ex-ante). RRI principles in this instance are translated as potential evaluation criteria.

In another variation in the same cluster, doing a pilot activity is translated within a network of RRI practitioners, PhD students, their supervisors, PhD committees and university management personnel. In this particular setting piloting is thought of as convincing “innovators” and further along the line a university administration of the usefulness of RRI-like approaches for PhD students.

“*And the whole idea was to, for us the idea was to show also to the researcher how like bringing Quadruple Helix involvement and citizen participation can really bring an added value to their project and to have a kind of case study or I don't know how to call it. Like an example or concrete case of, to be able to/ because I mean I know that the final aim (…) is to try to have an impact on policies et cetera but we wanted basically to have a concrete case of RRI applied to a research innovation project here (…).”*

This implies a particular theory of change: a theory of change that starts with the concrete things developed by researchers and thus also in the community of researchers and innovators, including universities.

“*So we [have] been saying that maybe there is some added value from including or having like citizen participation within the innovation process and the setup is meant to actually try to identify this. Because then it becomes practical and if it's something for the innovators that adds value, it does not need to be enforced by law. It comes from their own interest because they know it would be some kind of sustainability for their innovations.”*

The idea here is that by changing the perception of the innovators through the pilot activities, a long-term change in the cultures of research and innovation can be achieved. Hence, the aim is also to establish this as a standard activity within universities and PhD projects. Therefore, this is not only about convincing innovators about the added value of these engagements; it's also about convincing the universities and the doctoral program organizers.

However, there is also an awareness that “identifying” the added value always needs to come first. This indicates an awareness that there is no single “added value” for each and every R&I project, but that there might be different ones for different projects. Overall, then, RRI is translated in a double sense, as both a form of service to the broader R&I ecosystem as well as a form of analysis of pre-existing projects.

### Legacy and impact—RRI as maintenance work

Building on this, we also want to talk about how the relationship between project activities, outcomes, legacy and impact are thought of in the RRI projects we looked at.

The work with a regional university presents a quite clear idea about the legacy of the project activities and the impact of the work done. To start with, this idea is contrasted with a model of change through policy documents, something that is called “nice words” or “wishful thinking” and has been addressed in the literature as “buzzwords” by e.g., Vincent (2014).

“*It's quite a lot for an innovator, OK. So it should be working in two hours but, right or we don't know. And we have to understand because we are doing that, we are doing that as scientists. We want to objectify something. We want to go out of wishful thinking about the beauty of citizen engagement, OK. We cannot afford that. So each research project could be tested that way but then we have to be very efficient.”*

In contrast, the theory of change presented here focuses on working directly with innovators on the level of a PhD education. The impact of this could then be to initiate a cultural shift toward RRI with the next generations of innovators-in-training. This idea of having impact “on the ground” is in some ways similar but also distinct from the other pilot activities.

Another pilot activity in the same regional cluster takes a different approach toward RRI and works in the area of social innovations around the issue of unsold food in the Brussels-Capital region. Our colleagues work with different initiatives addressing food waste in Brussels, which ended up being in competition with each other. This pilot thus works very much “on the ground” and aims to have a more direct impact by helping to solve controversies and by supporting local government agencies to find ways of sustaining successful initiatives, while at the same time working toward improvements of evaluation criteria.

In contrast, the Lombardy cluster distinguishes so-called “preparatory activities” from the actual pilot activities. Preparatory activities being the collaborative development of the activities and the different instruments that are used in the activities (e.g., a survey or a focus group). In addition, the collaborative identification of the issues that should be addressed in the shared activity is part of this preparatory stage. The actual pilot activity, then, is the conduct of the planned agenda setting process; sending out the survey, analyzing the responses, holding a group discussion and so on.

The point we want to make here is that in terms of RRI and especially when it comes to questions of impact and legacy, both activities are equally important. The preparatory stage might be even more important because it is here institutional cultures of RRI can be influenced and shaped (if this is at all possible). It is also where pre-existing relations are cultivated, nurtured and maintained. These are activities that are concerned with maintenance and thus follow a logic of care. Importantly, this particular translation of RRI is premised on a longstanding relation between FGB and their regional administrative partners. The impact is not so much to convince this partner but rather to support them in spreading RRI principles within Lombardy Region.

Of course, these kinds of activities are difficult to measure and quantify. In order to do so one would have to ask actors from the regional government and administration partners how often they mention RRI or RRI-ish parts of their work to other colleagues. Additionally, and probably more importantly, these are activities that are not necessarily tied to the TRANSFORM project as such but are things that have been going on for quite some time and just now happen to take place with this project-frame. There is an interesting tension here between project temporalities and the maintenance or care work we observe here (for a similar observation in a different context see: Torka, 2006).

In the paragraph above we briefly alluded to the identification of issues, i.e., the collaborative process of defining what the activities should be about. This relates to the identification of so-called “windows of opportunity.” Differences in the institutional-organizational set-up in the different clusters are also expressed in how the clusters deal with windows of opportunity.

In the Brussels-Capital region a lot of work actually went into stabilizing the relationships between the different partners. As a consequence, it was more difficult to identify the exact windows of opportunity where collaborative RRI could be introduced in the work at the agency. In the conversations we had there was some talk of opportunities that might have been and that could be developed. It seems that through the initiative focused on unsold food such a window has been identified. In short, that window of opportunity may consist in a potential link between two stories about RRI, combining the two distinct objectives of establishing “meaningful” engagement in a civil society context, and participatory modes of ex-ante project evaluation within the government research funding agency. The work in this cluster thus might have created the conditions for future RRI activities by building a network and thus establishing a particular translation of RRI that is mutually beneficial for the different partners involved.

As a contrast, in the other regional clusters the collaboration between the different partners had already been more stable from the outset of the project. On the one hand, this steered the work in the clusters more toward maintenance and gradual extension which in turn makes it easier to identify fitting windows of opportunity. On the other hand, and this is a potential downside of such relations, there is always a risk of regulatory capture in the sense that there are instances when it is the administrative partner who has the power to define where RRI fits and where it doesn't. This is then framed as a pragmatic approach, and as being content with small successes.

### RRI and acceptance

When talking about RRI as a tool, an interesting question to ask is “what is it actually (imagined to be) a tool for?” We want to briefly point to one instance of such implicit objectives that came up in our work: different ideas related to the notion of “acceptance.” We say “interesting” because this is actually a rather tricky topic. However, that is exactly why we think it is worthwhile to spend a little time on this idea, on the role it plays in the accounts of the different cluster partners, and also how it relates to the broader RRI literature, where this term is rather contested when discussed as “acceptance politics” (Barben, 2010).

Before we come to that let's start with how this term is understood in the RRI literature. “Acceptability” has indeed been a part of conceptualizations of RRI from its very beginning, as visible in this quote from von Schomberg (2012):

“*Responsible Research and Innovation is a transparent, interactive process by which societal actors and innovators become mutually responsive to each other with a view to the (ethical) acceptability, sustainability and societal desirability of the innovation process and its marketable products (in order to allow a proper embedding of scientific and technological advances in our society).”*

Thinking about acceptance is in some ways similar to thinking about the contested issue of “trust” (Wynne, 2006). In some instances, for example when policymakers talk about trust, they might simply like citizens to trust in their ability to make well-informed, reasonable decisions for the public good. This will sometimes entail a discussion about the difficulty of gaining the trust of citizens. Another way of thinking about trust is in terms of decision-making or technological systems: for example, trust that there are systems in place, and that these systems allow for accountability and transparency.

When thinking about acceptance and acceptability in these terms, there are at least two versions. First, the more educationalist and awareness-raising understanding aims to inform and educate citizens so that they, in turn, will not reject technologies and policies on the basis of a lack of knowledge. The other version is to create processes through which citizens can actually influence the actual thing or decision, so that it is in fact more “accept-able”—this then is closer to the idea of RRI.

In the conversations we had about the cluster activities we addressed these nuances of the notion.

“*I mean for the moment (…) you have a campaign about air pollution but what will be the perception of the people that have been employed in the in the campaign and the people that have not been employed in the campaign. Is there a difference? If there is a decision for example to say okay, we will limit that thing to improve the quality of air, what would be the acceptance in the two different groups?”*

While this is a classic framing of the problem of acceptance, the notion also appears as the other of “social contestability” in the work with engineers. This conceptualization depicts citizens as a source for legitimate input; as a potential obstacle for innovation; and as an actor that is contesting what innovators or researchers are doing—albeit legitimately so in this account.

“*So they can focus on the technological aspect but they, they lack other competences that they could need in the lifecycle of, of the innovation at one moment. Sometimes very early, sometimes a bit later. And strangely, they are working like they hope that succeeding on the technological part will solve each and every problem. And we were asking them ‘but what about social contestability?' […] We discovered that we had to have a vehicle to a kind of, what we called at that time, one-stop shopping where they could find some competency that they don't have inside and that they could use that very early in the innovation process.”*

Here we see again a service logic attached to RRI work. RRI is understood as a “vehicle”—a tool—to deal with the problem of social contestability and create acceptance or possible conditions for acceptability. However, this is slightly different from the previous quote discussed above. The objective of bottom-up engagement here means helping the innovator “to anticipate some questions about their impact on the environment, on the public health, equity.”

## Conclusions—Tools and indicators for which kind of RRI?

We started this paper with a quote from Tim Ingold about tools, in which he directs attention to the question what makes a tool a tool. Talking about a mundane saw he argues that what lends the saw its “sawiness” are not inherent qualities or attributes but the story about its relationship to other things withing the given field of activity. In our case these are stories about how the RRI tools or participatory instruments enter into relationships with other things that constitute the specific context into which RRI is translated. We then proposed to ask what happens when a policy concept like Responsible Research and Innovation, understood as a collection of tools, born in the supra-national setting of the European Commission, is translated for purposes of science and innovation governance in regional settings. If a tool is its story, as Ingold put it, what are the stories of the RRI tools? How do the stories vary in the different territories and their respective R&I ecosystems?

To start with, in the cases we describe, RRI gets translated as a set of distinct approaches or methods, a toolkit of sorts. RRI takes on the shape of citizen science, shared agendas, participatory agenda setting, design thinking, or a citizens' jury. Notably, these are methods and tools that are well established beyond the scope of the RRI lemma, they are not specific to RRI and are not RRI-tools *per se*. Yet they are applied in a broader RRI framework. This means that they are used in a process of innovation governance in the broadest sense of the term.

What then are these methods a tool for? Here we see a great diversity of purposes and objectives. These range from regional urban development to attempts at re-shaping how innovation is conceptualized in the education of engineering PhDs, and from citizen science projects on health to introducing deliberative democracy into territorial innovation strategy development.

One important shared feature that in some ways draw this plurality of tools and purposes together, is the fact that the work that is being done mostly does not start from zero, but rather builds on pre-existing relationships and repertoires of collaboration. RRI as part of the SwafS funding program, then, is not necessarily a way to introduce new tools and concepts, but a way to continue ongoing work and follow pre-set purposes, aims and objectives. While these clearly correspond to RRI principles (often selectively so), what is happening in the different activities might be better understood as a form of “maintenance work.” What we mean by maintenance here is that existing relationships are cared for or re-kindled, networks are nurtured and further developed (this can be both by extension or by cutting unnecessary elements), and also methods and tools are adapted. In a similar manner Vinsel and Russell (2020, 15) in their work on technological innovation describe maintenance as “overlooked, undercompensated work and as a practice of “caring for the people and things that matter most to us, and ensuring that we preserve and sustain the inheritance of our collective pasts.” (ibid., 14f.) In this very human sense, RRI is deployed with a logic of care in the regional context, while the Horizon 2020 calls and proposals above all are formulated in a logic of choice, to be assessed by SMART indicators.

In all of this, the term RRI and its European lineage is doing very different things for different actors. The question then becomes what effects the ambition to develop something like RRI tools might have on the kind of work that we are seeing here. Here we would like to point out the following:

First, focusing on the development and standardization of tools for RRI can lead to a form of fragmentation. When RRI gets translated as method in an RRI ecosystem that needs tools to be easily applicable and replicable, the methods become the focus of the work. This can be seen in attempts at producing easy to follow guidelines and to create capacity within the regional administrations. While this clearly contributes to the ability of such approaches to “travel” (Czarniawska and Joerges, 2012) and shape the innovation cultures in different R&I ecosystems, the risk is that they are turned into “technologies of participation” (Soneryd, 2015) and the broader rationales and purposes the RRI and its predecessors strived for gets lost. When RRI becomes a method, it is very easy for R&I governance systems to lose sight of what they are actually methods for.

The second point we would like to make here relates back to the issue of maintenance work. Standardization processes either through indicator development or “toolification” might risk making this kind of work more difficult by inadvertently and in possibly unintended ways closing down certain forms of RRI that are not immediately recognizable without paying close attention. None of the achievements we have described in this paper, could have been detected by the MoRRI indicators as they were strictly defined by the MoRRI consortium (European Commission Directorate-General for Research Innovation et al., 2018) a similar conclusion may be drawn from the findings of Mehari et al. (2022). With some creative reinterpretation, perhaps the MoRRI indicators SLSE4, PE7 and GOV2 could be used to account for some results. By itself, that may not be so important. The problem arises, however, when regional actors depend on being able to demonstrate positive results to maintain their reputation and track record to secure further funding of their long-term maintenance work. In that case, and when indicator politics prevail, they may also feel the need to quantify and present indicator measurements. A related but broader phenomenon was pointed out by Sivertsen and Meijer (2020), in that funding bodies and evaluation practices give emphasis to achievements with high visibility, so-called “extraordinary impacts.” The risk is that maintenance and care work and more generally what the authors called ordinary impact, is given less appreciation and, ultimately, implicit discouragement.

Ultimately, work that focuses on nurturing relationships with the territorial R&I ecosystems in order to identify the most suitable ways of introducing RRI practices or work that aims at cultural change beyond the narrow limits of a single coordination and support action, runs the risk of getting sidelined and pushed to the margins at the expense of easily demonstrable and shiny impacts. If we take the ambition of RRI to “become mutually responsive to each other with a view to the (ethical) acceptability, sustainability and societal desirability of the innovation process and its marketable products” (von Schomberg, 2012) to enable “taking care of the future through collective stewardship of science and innovation in the present.” (2013, 1570) seriously, the dangers of toolification needs to be a part of ongoing reflections of RRI practices. We asked in the introduction how the performance of RRI should be evaluated in territorial contexts. We believe we have presented an argument against measurements against pre-set indicators and hence against benchmarking. Rather, by a slightly different route, we end up with the conclusion of de Rijcke et al. (2019), in favor of qualitative research and evaluative inquiry.

## Data availability statement

The datasets presented in this article are not readily available because. The dataset is confidential per recommendation of the Norwegian Social Science Data Services. Requests to access the datasets should be directed at: RS, roger.strand@uib.no.

## Ethics statement

Written informed consent was obtained from the individual(s) for the publication of any potentially identifiable images or data included in this article.

## Author contributions

TV analyzed materials, prepared the first draft of the manuscript, contributed to further editing, and approved of final version. MM contributed to further editing and approved of final version. RSl and RSt analyzed materials, contributed to further editing, and approved of final version. All authors contributed to the article and approved the submitted version.

## Footnotes

^1^https://digital-strategy.ec.europa.eu/en/library/rome-declaration-responsible-research-and-innovation-europe. Accessed May 23, 2022.

^2^Cf. e.g., the FP7-funded RRI Tools project (https://wbc-rti.info/object/project/14501; 2014–2016) and its objective to develop an RRI toolkit.

^3^Disclosure: One of the authors (Strand) chaired the Expert Group.

^4^https://ec.europa.eu/info/funding-tenders/opportunities/portal/screen/opportunities/topic-details/swafs-14-2018-2019-2020 https://ec.europa.eu/info/funding-tenders/opportunities/portal/screen/opportunities/topic-details/swafs-14-2018-2019-2020

^5^ https://super-morri.eu/rri-ecosystem/

^6^https://www.transform-project.eu/about-transform/. Accessed May 23, 2022.
